# A laboratory test to detect gliadin-specific CD4^+^ T-cells for difficult to diagnose celiac disease

**DOI:** 10.1016/j.jtauto.2025.100301

**Published:** 2025-07-24

**Authors:** Daan A.R. Castelijn, Nicolette J. Wierdsma, Kim de Buck, Maaike A. van Bree, Tracy-Jane T.H.D. Eisden, Jolien C. Hollander, Gerd Bouma, Hetty J. Bontkes

**Affiliations:** a Department of Laboratory Medicine, Laboratory Specialized Diagnostics and Research, Section Medical Immunology, Amsterdam Institute for Infection and Immunity, Amsterdam Gastroenterology Endocrinology Metabolism, Amsterdam University Medical Centers, Amsterdam, the Netherlands; b Department of Nutrition and Dietetics, Amsterdam Gastroenterology Endocrinology Metabolism, Amsterdam University Medical Centers, Amsterdam, the Netherlands; c Department of Gastroenterology and Hepatology, Amsterdam Gastroenterology Endocrinology Metabolism, Amsterdam University Medical Centers, Amsterdam, the Netherlands; d Department of Internal Medicine, St. Antonius Hospital, Nieuwegein, the Netherlands

**Keywords:** Celiac disease, Gluten free diet, Diagnosis, Gliadin-specific T-cells, Dextramers, Gluten challenge, Flow cytometry

## Abstract

**Objectives:**

Discrepancy between serology and small bowel histology, such as seronegative CD, poses a diagnostic challenge in celiac disease (CD) diagnosis. Recently described methods to detect gliadin-specific T-cells are too laborious even in a specialized diagnostic setting. We developed a method, which can be implemented in specialized diagnostic laboratories.

**Methods:**

Gliadin-specific T-cells were analyzed by α1-and α2-gliadin peptide loaded Dextramers (Dm) in healthy controls (HC, n = 18), patients with non-celiac gluten sensitivity (NCGS, n = 9), active CD (aCD, n = 7) and CD on a gluten free diet (GFD, n = 14). Control peptide (CLIP)-loaded Dm were used as background controls. The α-gliadin-Dm:CLIP-Dm ratio was calculated. In CD patients ≥5 years on GFD (n = 8), a randomized two-dose gluten challenge was performed to increase gliadin-specific T-cell frequencies.

**Results:**

Gliadin-specific CD4^+^ T-cell frequencies were significantly higher in aCD and GFD than in HC and NCGS (p ≤ 0.0001). In CD patients on a GFD ≥5 years, gliadin-specific T-cells were detectable in 6/8 patients after a week gluten challenge, and all tested positive within 4 weeks. Gliadin-specific T-cells significantly upregulated CD38 after 1 week of gluten ingestion (p < 0.008). Real world data from sixteen patients demonstrated the applicability of this test in diagnostic challenging cases.

**Conclusions:**

Gliadin-specific T-cells can be detected in peripheral blood of CD patients using commercially available dextramers. These cells persist in CD patients on a GFD but may decline over time. A short term low-dose gluten challenge increased sensitivity. This simplified detection method of gliadin-specific T-cells is suitable for diagnostic challenging CD cases.

## Highlights

• Dextramers can detect peripheral blood gliadin-specific CD4^+^ T-cells with a straightforward flowcytometry analysis method.
• Gliadin-specific T-cell frequency was significantly higher in CD patients compared to controls (p < 0.0001).
• A low-dose gluten challenge can increase gliadin-specific T-cells to detectable levels when undetectable pre-challenge.
• Gliadin-specific T-cell detection can aid in diagnosis of challenging clinical cases such as seronegative CD.

## 1 Introduction

Celiac disease (CD) is a chronic enteropathy caused by an inappropriate immune response to gluten peptides leading to duodenal lymphocytosis, crypt hyperplasia and villous atrophy [1]. IgA-antibodies against tissue transglutaminase (tTG-IgA) have a high accuracy for CD diagnosis [2,3], and are therefore commonly used in clinical practice as first-line screening tool [4]. In adults, histology of duodenal biopsies is performed to confirm diagnosis based on lymphocytosis and villous atrophy [3]. Once CD diagnosis is confirmed, patients are treated with a lifelong gluten-free diet (GFD). After introduction of a GFD, tTG-IgA antibodies will drop below detection level and intestinal damage will resolve [5,6]. However, particularly in adults, CD does not always present with high tTG-IgA titers and villous atrophy. Ambiguous results such as high anti-tTG IgA antibody titers without villous atrophy (potential CD) or lack of anti-tTG antibodies in patients with villous atrophy (seronegative CD, SnCD) can hamper prompt diagnosis and treatment. SnCD is estimated to have an incidence of 2–6.5 % but this may be underestimated because screening relies on serological testing [7]. Other CD entities presenting with low or no anti-tTG antibodies include ultrashort CD (UsCD) and CD patients on a self-initiated GFD. In UsCD patients, histological changes are not found in the second part of the duodenum but are limited to the duodenal bulb; these patients typically present with low anti-tTG antibody titers [8,9]. Self-initiation of a GFD is increasingly prevalent in individuals without confirmed CD diagnosis. In self-initiated GFD, it is important to distinguish true CD patients from non-CD individuals that suffer from intestinal or extra-intestinal symptoms related to ingestion of gluten, a disorder known as non-celiac gluten sensitivity (NCGS) [10]. GFD in non-CD individuals can cause health-risks as gluten-free products often contain less fibers and vitamins but more fat and sugar [11]. Furthermore, CD associated auto-immune diseases and complications such as iron deficiency and osteoporosis require additional testing and follow-up [12]. Current guidelines suggest a 2–6 weeks high dose (approx. 30 g/d) gluten challenge to re-induce serological and histological abnormalities. This is burdensome for patients, has poor compliance and low diagnostic accuracy [3,13,14]. For patients in which routine diagnostics are less suitable or fail, novel and non-invasive diagnostic strategies are needed to adequately and rapidly distinguish CD patients from individuals with other gluten-related complaints.

Presence of gliadin-specific T-cells is a hallmark of the immunopathogenesis of CD [15]. Gliadin proteins are main gluten components that induce proliferation of gliadin-specific CD4^+^ T-cells in the small intestine of CD patients. Previous studies demonstrated that these T-cells are detectable in peripheral blood of CD patients [16]. However, the current tetramer based protocol is laborious, necessitates enrichment of tetramer-positive cells and therefore precludes use in diagnostic settings [13]. Furthermore, there are no gliadin peptide loaded tetramers commercially available for diagnostic use. This study aimed to detect gliadin-specific T-cells using readily available Dextramers (Dm) with higher avidity compared to tetramers to develop a non-invasive blood test that can ultimately be used in specialized diagnostic settings for CD diagnosis.

Performance and diagnostic characteristics of Dm were investigated in a clinical study which included healthy controls (HC), disease controls and CD patients. In a proof-of-principle study we investigated whether a short low dose gluten challenge was sufficient to increase gliadin-specific T-cell frequency in CD patients on a GFD ≥5 years. Finally, we investigated the usefulness of the test in challenging cases with ambivalent serology and histology such as seronegative or potential CD in daily practice in a tertiary hospital.

## 2 Materials and methods

### 2.1 Study design and patients

A prospective single-center study was conducted from January 2020 until June 2023 at the Amsterdam University Medical Centers, a tertiary center in The Netherlands; see Supplemental Fig. 1A for study design. Potential adult participants suspected of active celiac disease (aCD), NCGS, known CD patients on GFD or HC were screened for HLA-DQ2.5; negative cases were excluded. Other exclusion criteria were no informed consent, immunosuppressive treatment during the past 3 months, seropositivity for HIV, hepatitis B/C or active malignancy. At inclusion peripheral blood gliadin-specific T-cells were measured as well as tTG-IgA and endomysium antibodies. HC without previous diagnosis of CD and using a gluten-containing diet were recruited among the hospital personnel and their friends and family. Unexpectedly, two HC had positive serology and were referred to their general practitioner and diagnosed with untreated active celiac disease (aCD). These participants were excluded. Diagnosis of aCD or NCGS was made after inclusion based on regular laboratory results and clinical evaluation. NCGS was defined by gastrointestinal symptoms occurring in relationship to gluten ingestion, but only after prior careful exclusion of a CD diagnosis, e.g. after gluten challenge. CD patients on GFD were included if their diagnosis was confirmed by serology and duodenal biopsy and a patient was on strict GFD >6 months. This group was subdivided in seronegative (tTG-IgA, <1xULN), and seropositive (tTG-IgA, >1x ULN). Laboratory personnel performing the dextramer analysis was blinded to the final diagnosis. In total 18 healthy controls, 9 NGCS and 21 CD patients were included.

A proof-of-principle gluten challenge was performed in newly recruited HLA-DQ2.5^+^ adult CD patients on a GFD ≥5 years. Patients were randomized to the 5g or 15g daily gluten group by the Electronic Data Capture Castor. Gliadin-specific T-cell frequency was determined weekly until at least one endpoint was met. The aim was to include 10 CD patients, but due to a paucity in recruitment, the study was closed when 8 CD patients were included. Inclusion criteria, study design and study endpoints are illustrated in Supplemental Fig. 1B.

Finally, in total 16 diagnostic challenging cases were tested off protocol with α1-Dm. A clinical diagnosis was made by the treating gastroenterologist based on extended diagnostic work-up. Gliadin-specific T-cell results were evaluated in these patients.

### 2.2 Serology

tTG-IgA was analyzed with the immunoassay EliA™ Celikey® IgA FEIA (Phadia AB, Thermo Fisher Scientific, Uppsala, Sweden). Testing was performed according to the manufacturer's instruction, and the recommended manufacturer cut-off was applied as a proxy for ULN (10 U/ml). Endomysium IgA-antibodies were analyzed by standardized indirect immune fluorescence tests [17].

### 2.3 HLA-DQ-gluten dextramers and flow cytometry analysis

To detect the presence of gliadin-specific T-cells, immunodominant α1-and α2-gliadin peptide loaded HLA-DQ2.5 Dextramers® (Immudex) were used. Class II-associated invariant chain peptide (CLIP) loaded HLA-DQ2.5 Dextramers® (Immudex) were used as background controls. Due to technical issues not all patients could be tested with α2-gliadin peptide loaded HLA-DQ2.5 Dextramers® (see Table 1).

**Table 1:** Characteristics of patients included.

	HC, n = 18	NCGS, n = 9^a^	aCD, n = 7^a^	CD on GFD^a^		CD on GFD ≥5 years and GC^a^	
				seropositive n = 5	seronegative n = 9	5g GC, n = 3	15g GC, n = 5
Male/female, n	5/13	1/8	4/3	5/9		0/3	3/2
Comorbidities	None	PCOS, breast cancer, curative	Vitiligo, eczema, hypothyroidism	Small bowel carcinoma, curative		None	Lactose intolerance
Mean duration on GFD, months (range)	0	13 (0–72)	0	41 (6–96)	113 (6–500)	155 (92–228)	173 (74–310)
March classification, n							
No biopsy	18	2	0	2	0	3	5
Marsh 0	0	7	0	1	3	0	0
Marsh 1	0	0	0	0	2	0	0
Marsh 2	0	0	1	0	0	0	0
Marsh 3A-C	0	0	6	2	4	0	0
tTG-IgA titer, n							
No serology	0	0	0	0	0	0	0
≤1 x ULN	18	9	0	0	9	3	5
>1x-≤3x ULN	0	0	0	0	0	0	0
>3x-≤10xULN	0	0	1	2	0	0	0
>10x ULN	0	0	6	3	0	0	0

CD = celiac disease, HC = healthy controls, NCGS = non celiac gluten sensitivity, aCD = active CD, GFD = gluten free diet, GC = gluten challenge, PCOS = polycystic ovary syndrome, tTG-IgA = tissue transglutaminase IgA, ULN = upper limit of normal.

a Due to technical reasons 11/18 HC, 1/9 NGCS, 1/7 aCD, 3/14 CD patients on GFD were not tested with the α2-Dm and two of the patients in the gluten challenge study were not tested with the α2-Dm at one or more follow-up points.

Absolute count of CD4^+^ T-cells was determined in peripheral blood mononuclear cells (PBMC) isolated from 36 ml heparinized blood by CD3, CD4, CD8 staining using trucount beads according to protocol (BD Biosciences). Washed PBMC (in PBS/5 % Fetal Calf Serum (FCS), GE Healthcare Life Sciences), equivalent to a maximum of 5x10^6^ CD4^+^ T-cells were incubated with APC-labelled and PE-labelled Dm (CLIP-, α1-gliadin- or α2-gliadin peptide loaded) for 30 min at room temperature in the dark. Subsequently cells were stained with fluorescent labelled monoclonal antibodies (25 min, 4–8 °C). Antibodies used: CD3-Alexa Fluor 700 (clone SP34-2), CD4-APC-H7 (clone SK3), CD45RA-BB515 (clone HI100), integrin-β7-BV421 (Iβ7, clone FIB283), CD38-BV711 (HIT2), all from BD Biosciences, CD45-Viogreen (clone 5B1, Miltenyi Biotec) and CD62L-PE-CY7 (clone DREG-56, BioLegend).

Cells were fixed (OptiLyse, Beckman Coulter according to manufacturer's instructions) and washed twice with PBS/5 % FCS. Cells were resuspended in PBS/5 % FCS and acquired using an Attune flowcytometer (ThermoFisher) equipped with acoustic focusing fluidics which enables high-sensitivity at fast sample flow-rates and high event rates. Data were analyzed using Kaluza Analysis Version 2.1 (Beckman Coulter).

### 2.4 α1- and α2-gliadin-specific T-cell clones

Clonal α1-gliadin (N10) and α2-gliadin (S4) peptide-specific CD4^+^ T-cells (courtesy of prof. F. Koning, Leiden University Medical Center) were cultured as previously described [18]. PBMC of healthy HLA-DQ2.5^+^ donors were spiked with these T-cell clones to confirm specificity of the α1-and α2-gliadin-loaded HLA-DQ2.5 Dm.

### 2.5 Statistics

Receiver operating characteristic (ROC) analysis, Mann-Whitney U and Wilcoxon signed rank sum tests were performed using GraphPad Prism Version 9.5 (GraphPad Software Inc.) and SPSS Version 22.0 (IBM SPSS Statistics). Data was collected using Electronic Data Capture Castor.

### 2.6 Ethical considerations

This study was approved by the local medical ethical committee of Amsterdam University Medical Centers (NL68731.029.19). Written informed consent was obtained from all participants.

## 3 Results

### 3.1 Performance of α1-and α2-gliadin peptide loaded Dm

**Fig. 1:**
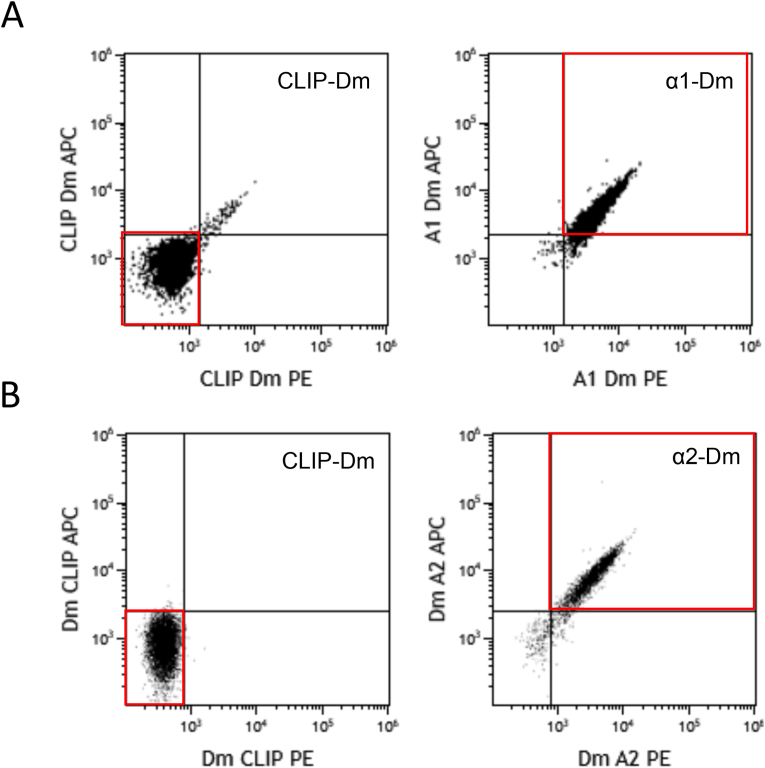
Expression of α1-, α2-and CLIP-Dm on gliadin-specific T-cell clones. (a) Expression of α1-Dm on α1-gliadin-specific T-cells (N10) after staining with fluorochrome-labelled α1-Dm (right) and negative control CLIP-Dm (left). (b) Expression of α2-Dm on α2-gliadin specific T-cells (S4) after staining with fluorochrome-labelled α2-Dm (right) and negative control CLIP-Dm (left).

The α1-and α2-gliadin peptide loaded HLA-DQ2.5 Dm showed staining with α1-and α2-gliadin specific CD4^+^ T-cell clones N10 and S4, respectively (Fig. 1A and B). Titration experiments with cell line N10 showed 10 μL α1-Dm per staining was the optimal volume as it resulted in the lowest background signal (Supplemental Fig. 2). Both α1-and α2-Dm were specific and no cross-reactivity between α1-Dm or α2-Dm was observed when tested with α1-and α2-gliadin specific CD4^+^ T-cell clones (Supplemental Fig. 3). Simultaneous combination of α1-and α2-Dm was feasible and did not show interference (Supplemental Fig. 4).

### 3.2 Diagnostic characteristics of α-gliadin peptide loaded Dm for celiac disease

**Fig. 2:**
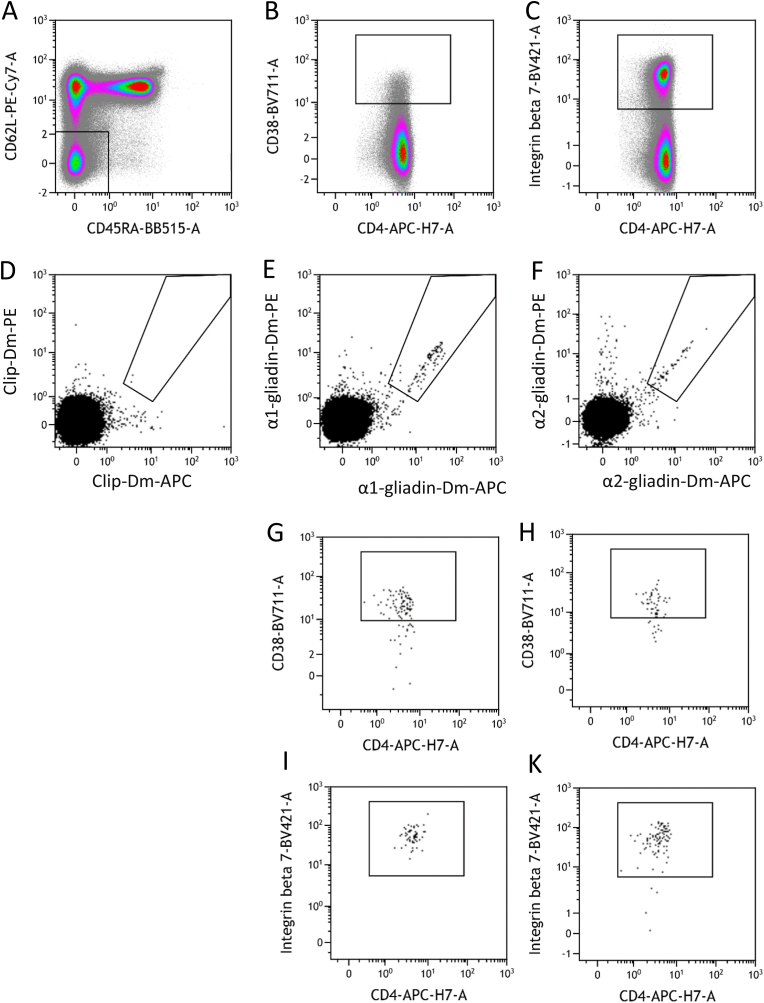
Gating strategy of a representative CD patient on GFD. CD4^+^ T-cell gated events during acquisition were saved. (a) CD62L and CD45RA double negative T_EM_ gated within CD4^+^ T-cells. (b) CD38 (c) Iβ7 expression within CD4^+^ T_EM_ (d) background staining with control CLIP-Dm within CD4^+^ T_EM_ (e) α1-gliadin- and (f) α2-gliadin-specific T-cells detected by PE and APC labelled Dm. CD38 (g, h) and Iβ7 (i, k) expression on α1-Dm (g, i) and α2-Dm (h, k) positive CD4^+^ T_EM._

**Fig. 3:**
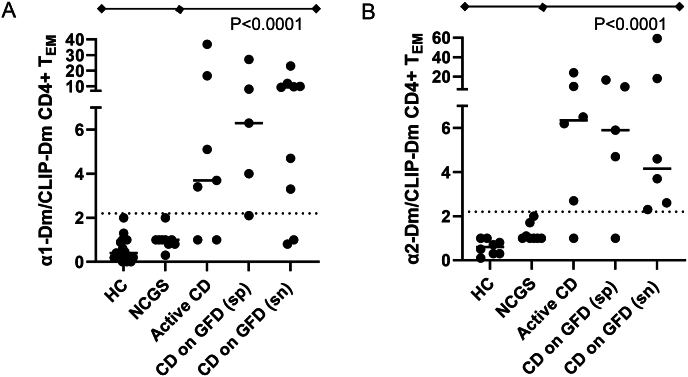
Ratios of α1-Dm:CLIP-Dm (a) and α2-Dm:CLIP-Dm (b) detected in the CD4^+^ T_EM_ cells circulating in the peripheral blood of different patient groups, including seropositive (sp) and seronegative (sn) CD patients on GFD. The dashed line illustrates the ROC derived cut-off value based on a specificity of 100 %.

Characteristics of study participants including comorbidities are shown in Table 1. No patients were on immunosuppressive therapies. A standardized protocol was developed to analyze PBMC of patients with CLIP-Dm, α1-Dm and α2-Dm. As gliadin-specific T-cells are expected to have an (effector) memory phenotype, expression of CD45RA and CD62L was determined to identify effector memory T-cells (T_EM_; CD45RA^−^, CD62L^−^), central memory (T_CM_; CD45RA^−^, CD62L^+^) and naive T-cells (T_N_; CD45RA^+^; CD62L^+^). Specific staining for α-gliadin-Dm was particularly present in T_EM_ (Supplemental Fig. 5A). A high background signal was mostly observed in T_CM_ and T_N_ (Supplemental Fig. 5B). Therefore, Dm^+^ T-cells were quantified for all patients within the T_EM_ population. To correct for variable background signals, a ratio of the α1-Dm or α2-Dm positive CD4^+^ T_EM_-cells and CLIP-Dm positive CD4^+^ T_EM_ was calculated. Fig. 2 shows a representative example of the analysis strategy. Double Dm positive events were considered relevant only if it was a compact population of at least 5 events. aCD and CD on GFD had significantly higher ratios for both α1-Dm and α2-Dm compared to NCGS patients and healthy controls (Fig. 3, p < 0.0001). Among the different CD groups (aCD, GFD and seronegative (sn), GFD and seropositive (sp)), there was no significant difference in Dm ratios.

Receiver operating characteristic (ROC) curve analysis based on disease controls (NCGS, n = 9) and all CD patients (n = 21) showed a high area under the curve (AUC) for both α1-Dm and α2-Dm:CLIP ratios (≥0.90; p ≤ 0.0009, Supplemental Fig. 6A and 6C). Using cut-off values based on ROC curve analysis (>2.2) and high specificity (100 %) (Supplemental Fig. 6B and 6D), α1-gliadin-specific T-cells were detected in 16/21 tested CD patients (76 %) and α2-gliadin-specific T-cells were detected in 15/17 tested CD patients (88 %) (Fig. 3A and B). In total 18/21 (86 %) of CD patients were positive for α1-gliadin and/or α2-gliadin-specific T-cells.

To illustrate the actual frequency of gliadin-specific T-cells, correction for background was also done by subtracting the number of CLIP-Dm positive cells from the number of α1-Dm and α2-Dm positive cells (Supplemental Fig. 7A and 7B), showing a frequency of 0–37 α1-gliadin-specific T-cells and 1–59 α2-gliadin-specific T-cells per million CD4^+^ T-cells in the CD groups.

### 3.3 Randomized two-dose proof of principle gluten challenge

**Fig. 4:**
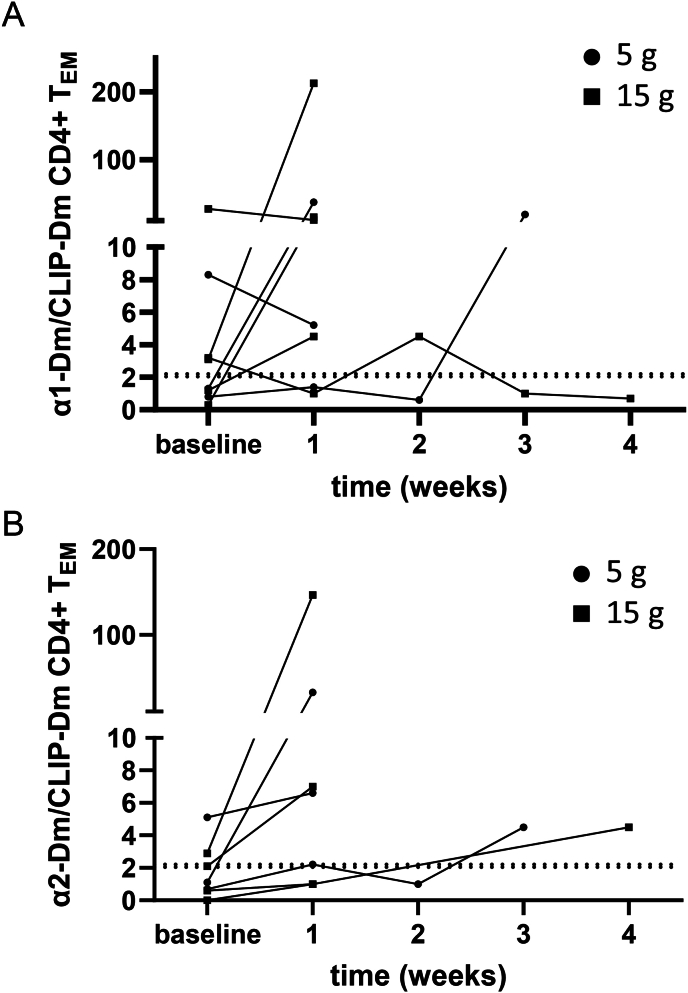
Detection of α-gliadin-specific T-cells in peripheral blood with Dextramers after a randomized gluten challenge (GC) accurately identifies celiac disease patients on a gluten-free diet. (a) α1-Dm:CLIP-Dm and (b) α2-Dm:CLIP-Dm ratios in CD4^+^ T_EM_ at baseline and after randomized two-dose gluten challenge in CD patients (n = 8). 2 patients were not tested with α2-Dm due to temporary unavailability of the reagent. The dashed line illustrates the cut-off.

Because it is expected that gliadin-specific T-cell frequencies decline below detection levels over time, we included patients that had been on a GFD for at least 5 years (Table 1) for the gluten challenge. Indeed, 50 % of the patients were negative for gliadin-specific T-cells at baseline compared to only 14 % of CD patients in the cross-sectional part of this study. After only one week of gluten challenge, 6/8 and 4/6 patients showed α1-Dm or α2-Dm:CLIP ratios higher than the cut-off value (Fig. 4A and B). One patient responded after three weeks with both α1-and α2-gliadin Dm. One patient responded only with α2-Dm. Due to technical reasons this patient was first tested for α2-Dm at week 4. It is therefore unknown if this patient responded at an earlier timepoint. As expected, tTG-IgA and endomysium-IgA antibodies remained negative for all CD patients during the gluten challenge (data not shown). Altogether, all 8 CD patients on a GFD tested positive after gluten challenge, including the three patients in the 5g gluten dose group. The rate of ratio increase was independent of gluten dose.

### 3.4 CD38 expression

**Fig. 5:**
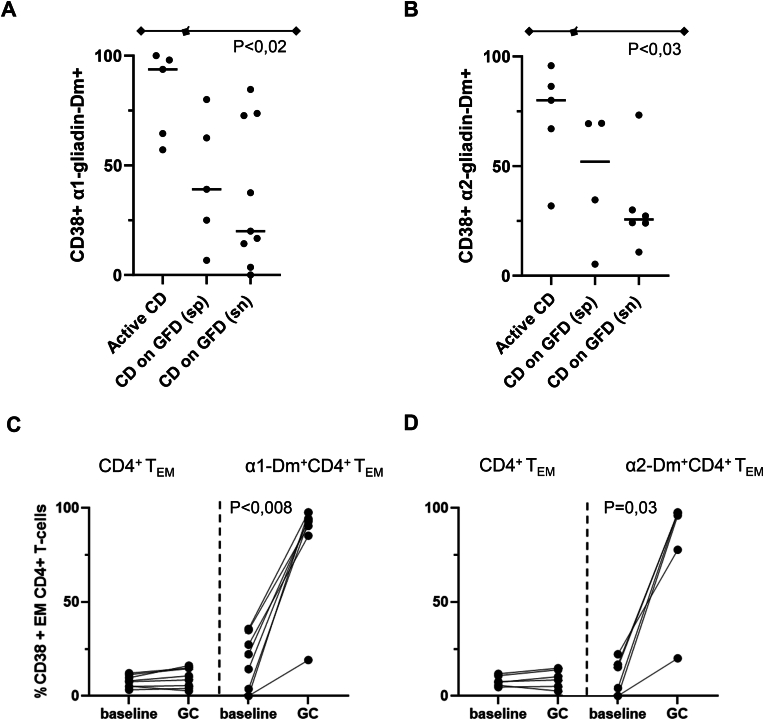
CD38 expression on α1-Dm (a) and α2-Dm (b) positive cells are shown in aCD and CD patients on GFD. Horizontal lines indicate the median. Percentage CD38 positive α1-Dm (c) and α2-Dm (d) positive and negative CD4^+^ T_EM,_ at baseline and 1 week after gluten challenge.

Previous studies have shown CD38 and integrin-β7 on gluten-specific T-cells and selective upregulation of CD38 on gliadin-specific T-cells after gluten challenge [13,19,20]. Here we confirm that the majority of the gliadin-specific T-cells expressed integrin-β7 showing mucosal origin; evaluation of this marker did not add to the specificity of the assay (data not shown). In addition, the activation marker CD38 showed significantly higher expression on gliadin-specific T-cells in aCD patients compared to CD patients on GFD (Fig. 5A and B). A trend towards lower CD38 expression on gliadin-specific T-cells was observed in seronegative GFD patients compared to seropositive patients. Furthermore, the percentage of CD38-positive gliadin-specific T-cells increased significantly after 1 week of gluten challenge compared to baseline (p < 0.008 and p = 0.03; Wilcoxon signed rank sum test for α1-gliadin and α2-gliadin specific T-cells respectively), whereas CD38 expression on other T_EM_ remained low (Fig. 5C and D).

### 3.5 Off protocol testing in challenging patients: real world data

In total 16 patient suspected for CD with discrepancies in clinical signs, serology, histology and/or response to GFD or GC were tested off-protocol for α1-Dm. These patients were referred countrywide to Amsterdam University Medical Centers, the national celiac disease expertise center. Ten were on a GFD and were asked to take a one week GC prior to testing for gliadin-specific T-cells, five refused a GC (Table 2). Four patients were diagnosed with SnCD based on the following clinical and diagnostic parameters: other causes of villous atrophy (such as medication, infection, autoimmunity, CVID, AIE, IBD) were excluded in all cases; increased percentage gamma delta T-cells among IEL in two cases (not tested in two cases); a clinical response upon a GFD in all four cases; in two cases histological response was confirmed (other two cases have had no follow-up biopsy (yet)). Three SnCD patients had clearly detectable levels of gliadin-specific T-cells whereas one had a value just above the cut-off level. Three patients were diagnosed with potential CD, all cases were positive for gliadin-specific T-cells, demonstrating classical immune-mediated CD. Four patients with low antibody titers (<3xULN) and villous atrophy were clinically diagnosed with CD; all had detectable gliadin-specific T-cells. Four patients had gluten related gastrointestinal and/or neurological symptoms and first degree family member(s) with proven celiac disease and were diagnosed as NCGS; three tested negative, one had a value just above the cut-off level. In one patient with longstanding villous atrophy but without gluten related symptoms CD was excluded but diagnosis remains unknown; this patient tested negative (Table 2).

**Table 2:** Characteristics of the patients tested off protocol.

	Seronegative CD n = 4	Potential CD n = 3	CD n = 4	NCGS n = 4	Unknown n = 1
Mean duration on GFD, months (range)	16 (0–48)	4 (0–13)	31 (3–72)	50 (0–180)	72
Eats gluten	2	2	1	1	0
One week GC	1	1	2	1	0
Gluten free	1	0	1	2	1
Marsh classification, n					
No biopsy	0	0	0	3	0
Marsh 0	0	3	0	1	0
Marsh 1	0	0	1	0	0
Marsh 2	0	0	0	0	0
Marsh 3A-C	4	0	3	0	1
tTG-IgA titer, n					
No serology	0	0	1	0	0
≤1 x ULN	4	0	0	4	1
>1x-≤3x ULN	0	2	3	0	0
>3x-≤10xULN	0	0	0	0	0
>10x ULN	0	1	0	0	0
Gliadin specific T-cell result, n					
Negative	0	0	0	3	1
Equivocal	1	0	0	1	0
Positive	3	3	4	0	0

## 4 Discussion

Our study demonstrated that the use of Dextramers to detect gliadin-specific T-cells is feasible in a specialized diagnostic laboratory and that it may aid to identify CD patients when serology and small intestinal histology show discrepancies. This can support gastroenterologists to diagnose CD less invasively in challenging cases such as seronegative CD as demonstrated by the real world data. This approach is patient friendly compared to current high dose and long term gluten challenge protocols.

Previous studies have investigated the value of gliadin-specific T-cells to diagnose CD [13,19]. Based on a HLA-DQ-gluten tetramer-based assay, the sensitivity was 97 % and specificity was 95 % for CD on GFD versus NCGS [21]. These results shown in larger groups are similar to our findings. However, the described test using tetramers requires a pre-enrichment step to reach sufficient sensitivity for detection of the low frequent specific T-cells. Furthermore, as far as we know gliadin peptide loaded tetramers are not commercially available. Dm are readily available and have higher avidity to bind antigen-specific T-cells compared to tetramers, making it easier to detect low-count T-cells [[22], [23], [24]]. As such, use of Dm allows avoidance of cumbersome enrichment steps, optimizing workflow and facilitating implementation in a specialized diagnostic setting. Laboratories that have access to flow cytometers with an acquisition speed of 20.000–30.000 events per second will be able to efficiently perform this novel Dm-based diagnostic test on 2–3 samples within 5–6 h.

Five out of 14 CD on GFD patients had detectable tTG-IgA levels, this can either be due to a slow reduction in tTG-IgA levels to below the diagnostic cut-off which can take years, or due to inadvertent gluten intake [6]. There is however no significant difference in the gliadin-specific T-cell ratios between tTG-IgA positive and negative patients. It is conceivable that the frequency of circulating gliadin-specific T_EM_-cells decreases over time. And indeed, gliadin-specific T-cells were not detected in all CD patients on a GFD. Therefore we investigated whether a short low dose of gluten was sufficient to increase the proportion of gliadin-specific T-cells in patients on GFD ≥5 years. Indeed, the detection threshold was exceeded in all tested CD patients within 4 weeks and in the majority of patients already after only one week even with a very low gluten dose of 5g/day. These data demonstrate that CD can be diagnosed after a shorter, less burdensome gluten challenge as compared to the standard 6-week challenge for conventional diagnosis, similar to the previously reported gliadin-specific T-cell detection with tetramers after a 14-day gluten challenge [13].

Additionally, other T-cell immunoreactivity assays to gluten in peripheral blood are being investigated, such as IL-2 release after in vivo gluten challenge [25] or in vitro gluten stimulation [26,27]. In our study, serum IL-2 concentration was measured before and 4 h after first gluten challenge. Six out of eight CD patients on GFD showed significant IL-2 concentration increase (data not shown); all HC, including the 2 HC that were later determined to be aCD patients, did not demonstrate any IL-2 kinetics after gluten ingestion. Recently it was demonstrated in a large cohort that whole blood IL-2 production after gluten peptide stimulation accurately reflects T-cell immunoreactivity to gluten and is a sensitive and specific biomarker [27]. In this study by Moscatelli et al., two aCD patients also demonstrated no plasma IL-2 increase after in vitro gluten stimulation (similar to the two HC who had aCD in our gluten challenge study), however showed positive response when the test was repeated after starting GFD. Possibly chronic gluten-induced T-cell stimulation can impair the gluten induced IL-2 response. Using IL-2 as a marker for T-cell immunoreactivity after in vitro gluten stimulation is promising, particularly in patients already on GFD, but has not yet been studied in more difficult to diagnose CD patients such as snCD or potential CD.

Gluten related activation of the specific T-cells was also demonstrated in our study by increased CD38 expression in both aCD patients and the gluten free patients that were challenged, which is in agreement with previous data [20,21]. Interestingly five patients in the CD on GFD group showed high (>50 %) CD38 expression without gluten challenge irrespective of seropositivity, possibly indicating (hidden) gluten intake. This feature may help to detect hidden gluten intake in patients that remain symptomatic on GFD. The combined detection of gliadin-specific CD4^+^ T-cells and gluten related CD38 expression is a promising addition to the diagnostic tool kit for challenging CD cases. The test has particularly a high positive predictive value, as all positive subjects were diagnosed with CD, including the off-protocol patients. The negative predictive value in this small study is 81 %, as four CD patients, including two aCD patients, had undetectable or equivocal α1 and α2-gliadin-specific CD4^+^ T-cells. Further improvement of sensitivity of the assay may be achieved by the inclusion of additional immunogenic gliadin-peptides such as γ- and ω-gliadins which have been included in the previous tetramer studies [19,21].

Next to the small sample size, a limitation of our study is that the utilized Dm only detect HLA-DQ2.5 restricted T-cells, but not HLA-DQ8 and HLA-DQ2.2 restricted T-cells. Approximately 90 % of CD patients are HLA-DQ2.5^+^ and thus constitute the majority of cases, but the remainder can currently not be tested [28]. Therefore, future studies should include HLA-DQ8 and HLA-DQ2.2 Dm.

In conclusion, we show that Dextramers to detect α-gliadin-specific CD4^+^ T_EM_ can accurately identify most celiac disease patients irrespective of the use of a (self-initiated) GFD and that this test is methodologically feasible in a specialized laboratory setting. Validation in larger cohorts should demonstrate its potential in diagnostic practice. In CD patients on GFD, a short, low dose gluten challenge is sufficient to increase CD38^+^ activated gliadin-specific T-cells and establish CD diagnosis. Data from this study show that the Dm test is a promising additional diagnostic tool, particularly in cases where serology is not informative such as snCD, but potentially also in ultra short CD.

## CRediT authorship contribution statement

**Daan A.R. Castelijn:** Writing – original draft, Visualization, Validation, Methodology, Investigation, Formal analysis, Data curation. **Nicolette J. Wierdsma:** Writing – review & editing, Visualization, Validation, Supervision, Methodology, Investigation, Funding acquisition, Formal analysis, Data curation, Conceptualization. **Kim de Buck:** Writing – review & editing, Visualization, Validation, Project administration, Methodology, Investigation, Data curation. **Maaike A. van Bree:** Writing – review & editing, Validation, Investigation, Data curation. **Tracy-Jane T.H.D. Eisden:** Validation, Investigation, Formal analysis, Data curation. **Jolien C. Hollander:** Writing – review & editing, Validation, Investigation, Formal analysis, Data curation. **Gerd Bouma:** Writing – review & editing, Validation, Supervision, Methodology, Investigation, Formal analysis, Data curation, Conceptualization. **Hetty J. Bontkes:** Writing – review & editing, Writing – original draft, Visualization, Validation, Supervision, Resources, Methodology, Investigation, Funding acquisition, Formal analysis, Data curation, Conceptualization.

## Funding

This study was supported by a grant from the 10.13039/501100008359Maag Lever Darm Stichting (MLDS; D18-08) and the Nederlandse Coeliakie Vereniging (Dutch Celiac Disease Patient Society; NCV; personal grant NW). The funders had no role in the design and conduct of the study.

## Declaration of competing interest

The authors declare no potential competing interests.

## Data Availability

Data will be made available on request.

## Appendix A Supplementary data

Multimedia component 1 Fig. S1 (a)Study design and inclusion investigating the performance of Dextramer based detection of peripheral blood gliadin-specific T-cells for celiac disease diagnosis. (b) Study design and inclusion of side study demonstrating a proof-of-principle randomized gluten challenge Multimedia component 1

Multimedia component 2 Fig. S2Titration experiments with α1-Dm. White blood cells of an HLA-DQ2.5^+^ healthy donor were spiked with gliadin α1-specific clonal T-cells (N10). Cells were stained with CD4-APC-H7, CD3-Alexa Fluor 700 and different volumes of Dm (10μl, 20μl or 30μl each per staining), according to protocol. Data of the approximately 400.000 gated CD4^+^ T-cells in each condition are shown Multimedia component 2

Multimedia component 3 Fig. S3Specificity of α1-Dm and α2-Dm. The gliadin α1 (N10) and gliadin α2 (S4) specific T-cell clones were stained with CLIP-Dm, α1-Dm and α2-Dm Multimedia component 3

Multimedia component 4 Fig. S4Combination of α1-Dm and α2-Dm. 5 x 10^6^ PBMC of an HLA-DQ2.5^+^ healthy donor were spiked with 300 gliadin α1-specific clonal T-cells (N10), and/or 300 gliadin α2-specific clonal T-cells (S4). Cells were stained with CD4-APC-H7, CD3-Alexa Fluor-700 and gliadin α1 and/or gliadin α2 peptide loaded Dm Multimedia component 4

Multimedia component 5 Fig. S5A representative example of a CD patient on GFD with low or high background signal in the CLIP-Dm analysis. (a) GFD patient with low background Clip-Dm staining and detectable α1-gliadin-specific T-cells in the effector memory T-cell gate (EM). (b) GFD patients with high background Dm staining in central memory (CM) and Naïve (N) T-cell gates in both Clip-Dm and α1-gliadin Dm staining. E=effector T-cell gate Multimedia component 5

Multimedia component 6 Fig. S6Receiver operating characteristic (ROC) curve analysis of (a) and (b) α1- and (c) and (d) α2-Dm:CLIP-Dm ratios in CD4^+^ T_EM_ for diagnosis of CD Multimedia component 6

Multimedia component 7 Fig. S7The number of (a) α1-Dm positive and (b) α2-Dm positive CD4^+^ T_EM_, corrected for background by subtracting the number of CLIP-Dm positive CD4^+^ T_EM_. Open symbols illustrate individual patients with a ratio below the threshold Multimedia component 7
