# Supplementary material for: A laboratory test to detect gliadin-specific CD4+ T-cells for difficult to diagnose celiac disease
Source: J Transl Autoimmun. 2025 Jul 24;11:100301. doi: 10.1016/j.jtauto.2025.100301 (PMC12329281; doi:10.1016/j.jtauto.2025.100301)
Supplement: Multimedia component 1 — Fig. S1 (a)Study design and inclusion investigating the performance of Dextramer based detection of peripheral blood gliadin-specific T-cells for celiac disease diagnosis. (b) Study design and inclusion of side study demonstrating a proof-of-principle randomized gluten challenge [file mmc1.pptx]

## Slide 1
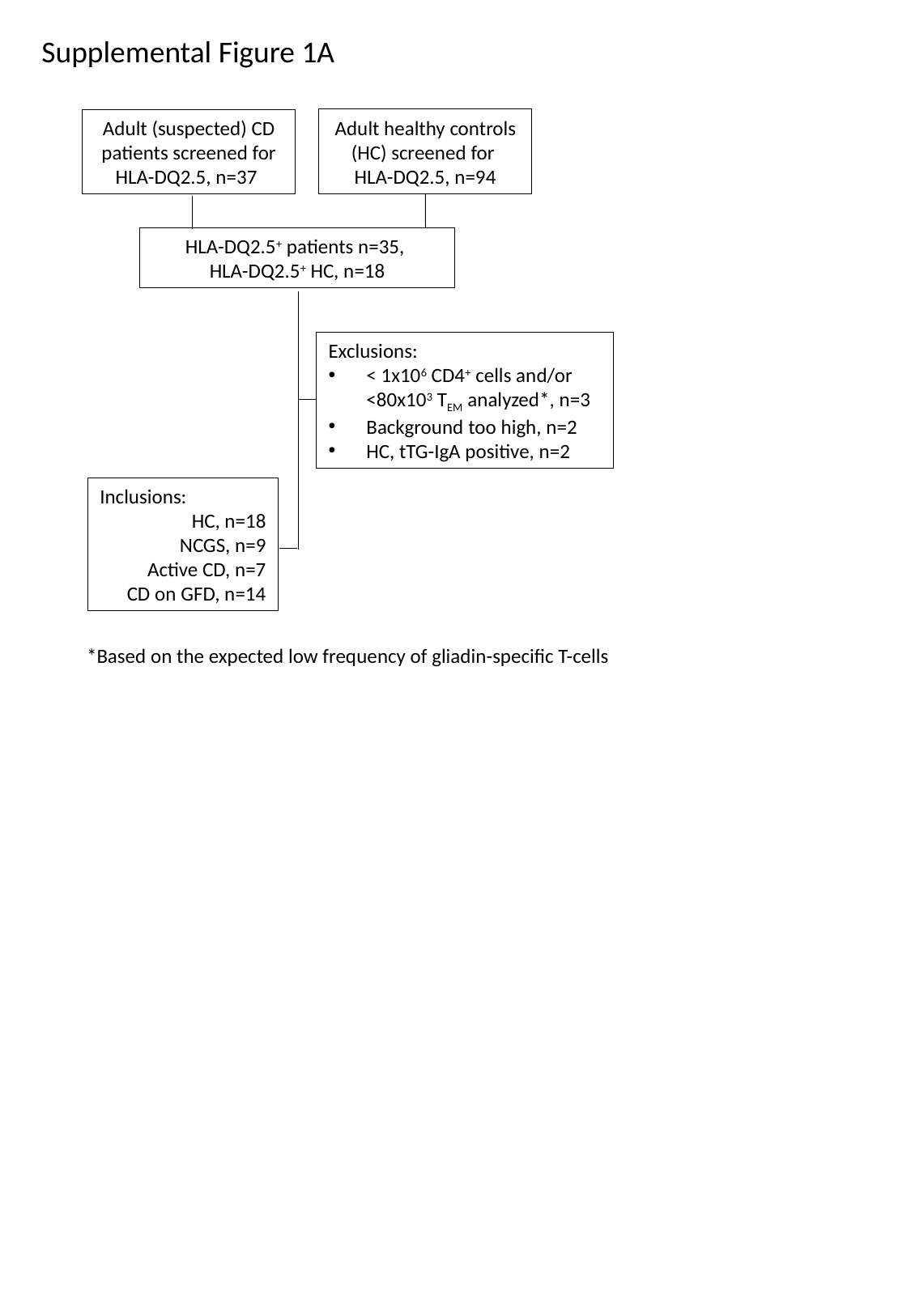

Supplemental Figure 1A
Adult healthy controls (HC) screened for
HLA-DQ2.5, n=94
Adult (suspected) CD patients screened for HLA-DQ2.5, n=37
HLA-DQ2.5+ patients n=35,
HLA-DQ2.5+ HC, n=18
Exclusions:
< 1x10^6^ CD4+ cells and/or <80x103 TEM analyzed*, n=3
Background too high, n=2
HC, tTG-IgA positive, n=2
Inclusions:
HC, n=18
NCGS, n=9
Active CD, n=7
CD on GFD, n=14
*Based on the expected low frequency of gliadin-specific T-cells

## Slide 2
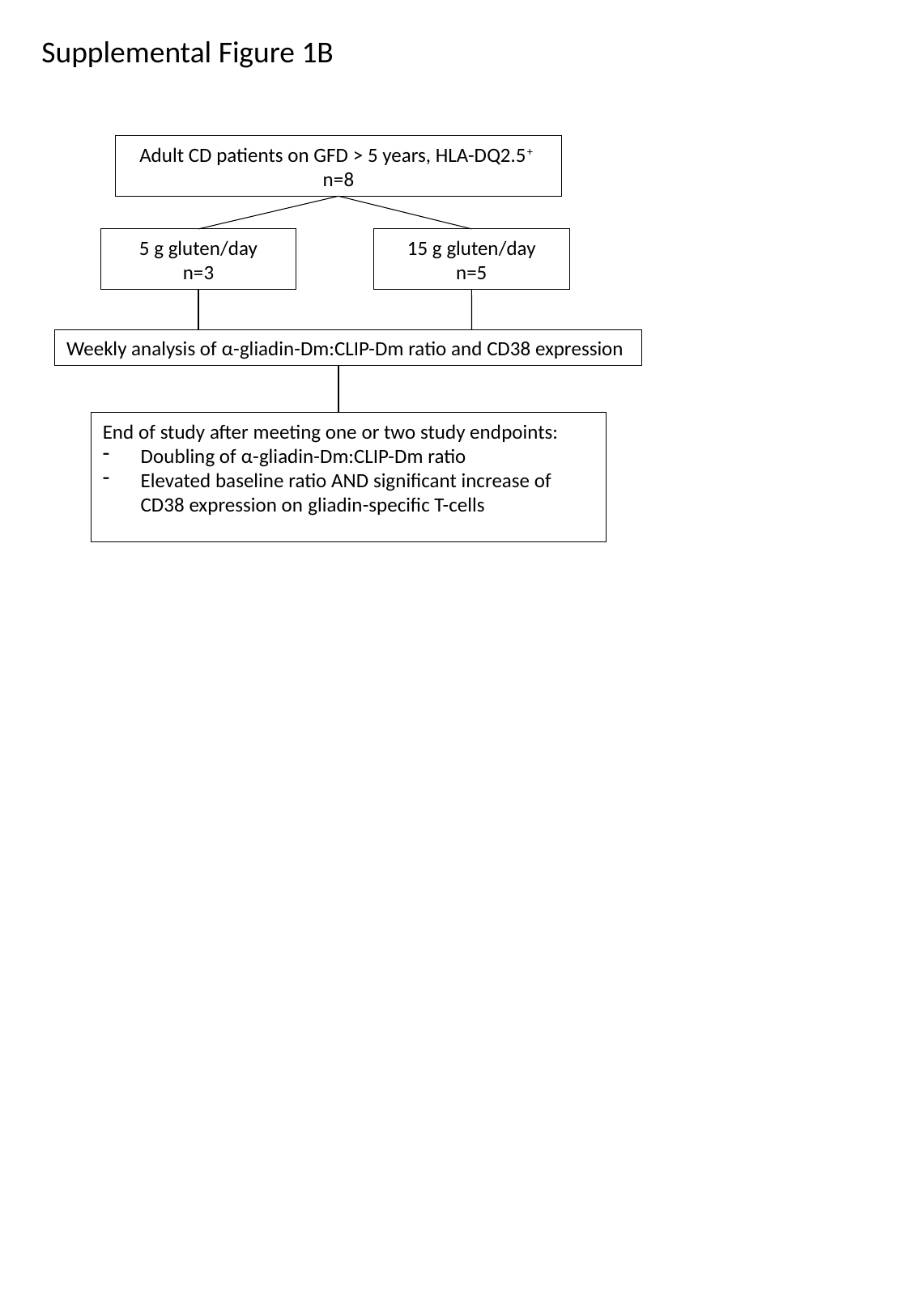

Supplemental Figure 1B
Adult CD patients on GFD > 5 years, HLA-DQ2.5+ n=8
5 g gluten/day
n=3
15 g gluten/day
n=5
Weekly analysis of α-gliadin-Dm:CLIP-Dm ratio and CD38 expression
End of study after meeting one or two study endpoints:
Doubling of α-gliadin-Dm:CLIP-Dm ratio
Elevated baseline ratio AND significant increase of CD38 expression on gliadin-specific T-cells
