# Supplementary material for: A laboratory test to detect gliadin-specific CD4+ T-cells for difficult to diagnose celiac disease
Source: J Transl Autoimmun. 2025 Jul 24;11:100301. doi: 10.1016/j.jtauto.2025.100301 (PMC12329281; doi:10.1016/j.jtauto.2025.100301)
Supplement: Multimedia component 2 — Fig. S2Titration experiments with α1-Dm. White blood cells of an HLA-DQ2.5+ healthy donor were spiked with gliadin α1-specific clonal T-cells (N10). Cells were stained with CD4-APC-H7, CD3-Alexa Fluor 700 and different volumes of Dm (10μl, 20μl or 30μl each per staining), according to protocol. Data of the approximately 400.000 gated CD4+ T-cells in each condition are shown [file mmc2.docx]

**Supplemental Figure 2.**


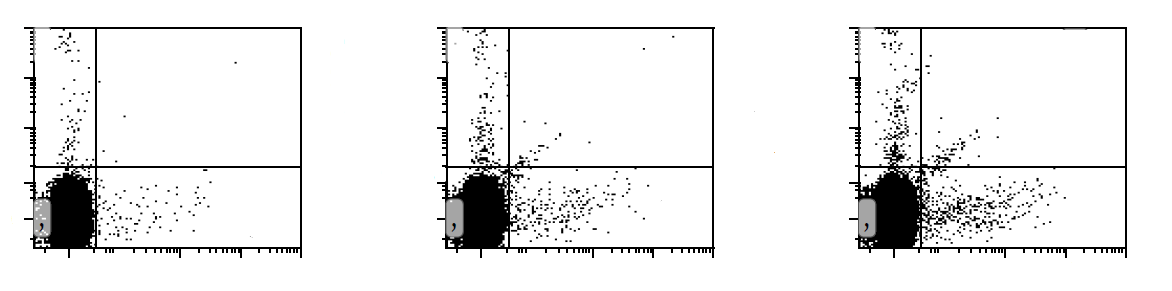

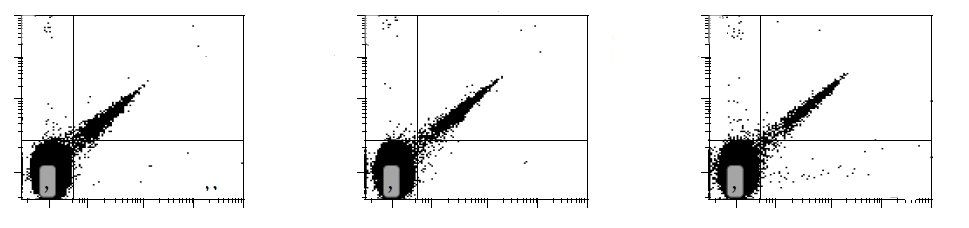


CLIP Dm-PE

CLIP Dm-APC

Gliadin α1 Dm-APC

Gliadin α1 Dm-PE

10µl

20µl

30µl

0.00%

0.01%

0.02%

0.33%

0.40%

0.30%

CLIP Dm-APC

CLIP Dm-PE

CLIP Dm-APC

CLIP Dm-PE

Gliadin α1 Dm-APC

Gliadin α1 Dm-APC

Gliadin α1 Dm-PE

Gliadin α1 Dm-PE
