# Supplementary material for: A laboratory test to detect gliadin-specific CD4+ T-cells for difficult to diagnose celiac disease
Source: J Transl Autoimmun. 2025 Jul 24;11:100301. doi: 10.1016/j.jtauto.2025.100301 (PMC12329281; doi:10.1016/j.jtauto.2025.100301)
Supplement: Multimedia component 3 — Fig. S3Specificity of α1-Dm and α2-Dm. The gliadin α1 (N10) and gliadin α2 (S4) specific T-cell clones were stained with CLIP-Dm, α1-Dm and α2-Dm [file mmc3.docx]

**Supplemental Figure 3.**


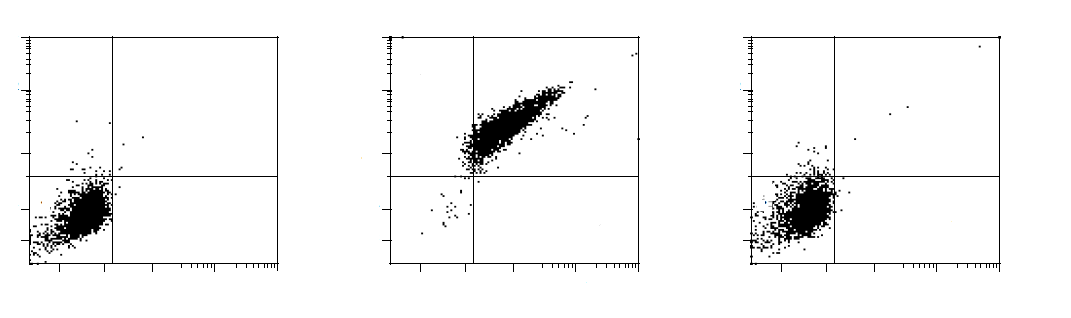


CLIP Dm-PE

Gliadin α1 Dm-APC

CLIP Dm-APC

Gliadin α1 Dm-PE

Gliadin α2 Dm-APC

Gliadin α2 Dm-PE

Gliadin α1 specific T-cell clone (N10)


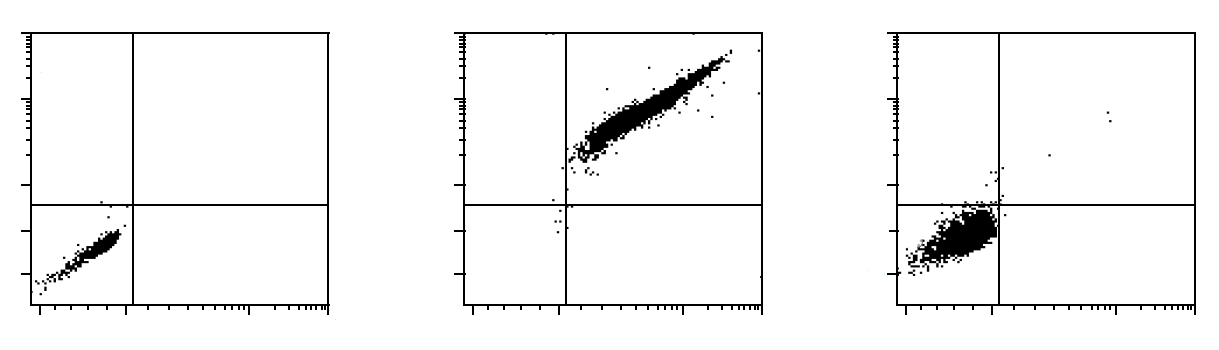


CLIP Dm-PE

Gliadin α2 Dm-APC

CLIP Dm-APC

Gliadin α2 Dm-PE

Gliadin α1 Dm-APC

Gliadin α1 Dm-PE

Gliadin α2 specific T-cell clone (S4)
