# Supplementary material for: A laboratory test to detect gliadin-specific CD4+ T-cells for difficult to diagnose celiac disease
Source: J Transl Autoimmun. 2025 Jul 24;11:100301. doi: 10.1016/j.jtauto.2025.100301 (PMC12329281; doi:10.1016/j.jtauto.2025.100301)
Supplement: Multimedia component 4 — Fig. S4Combination of α1-Dm and α2-Dm. 5 x 10^6^ PBMC of an HLA-DQ2.5+ healthy donor were spiked with 300 gliadin α1-specific clonal T-cells (N10), and/or 300 gliadin α2-specific clonal T-cells (S4). Cells were stained with CD4-APC-H7, CD3-Alexa Fluor-700 and gliadin α1 and/or gliadin α2 peptide loaded Dm [file mmc4.docx]

**Supplemental Figure 4.**


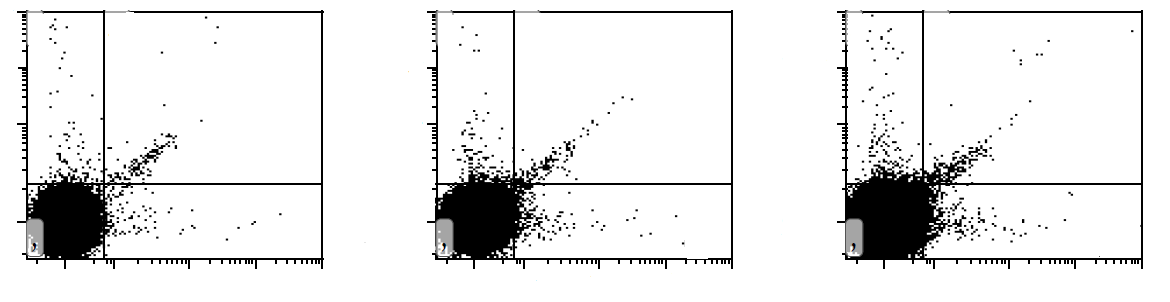


Gliadin α1 specific T-cell clone (N10)

Gliadin α1 Dm-PE

Gliadin α1 Dm-APC

Gliadin α2 Dm-PE

Gliadin α2 Dm-APC

Gliadin α1 and α2 Dm-PE

Gliadin α1 and α2 Dm-APC

Gliadin α2 specific T-cell clone (S4)

N10 and S4

0,01%

0,02%

0,03%
