# Supplementary material for: A laboratory test to detect gliadin-specific CD4+ T-cells for difficult to diagnose celiac disease
Source: J Transl Autoimmun. 2025 Jul 24;11:100301. doi: 10.1016/j.jtauto.2025.100301 (PMC12329281; doi:10.1016/j.jtauto.2025.100301)
Supplement: Multimedia component 5 — Fig. S5A representative example of a CD patient on GFD with low or high background signal in the CLIP-Dm analysis. (a) GFD patient with low background Clip-Dm staining and detectable α1-gliadin-specific T-cells in the effector memory T-cell gate (EM). (b) GFD patients with high background Dm staining in central memory (CM) and Naïve (N) T-cell gates in both Clip-Dm and α1-gliadin Dm staining. E=effector T-cell gate [file mmc5.pptx]

## Slide 1
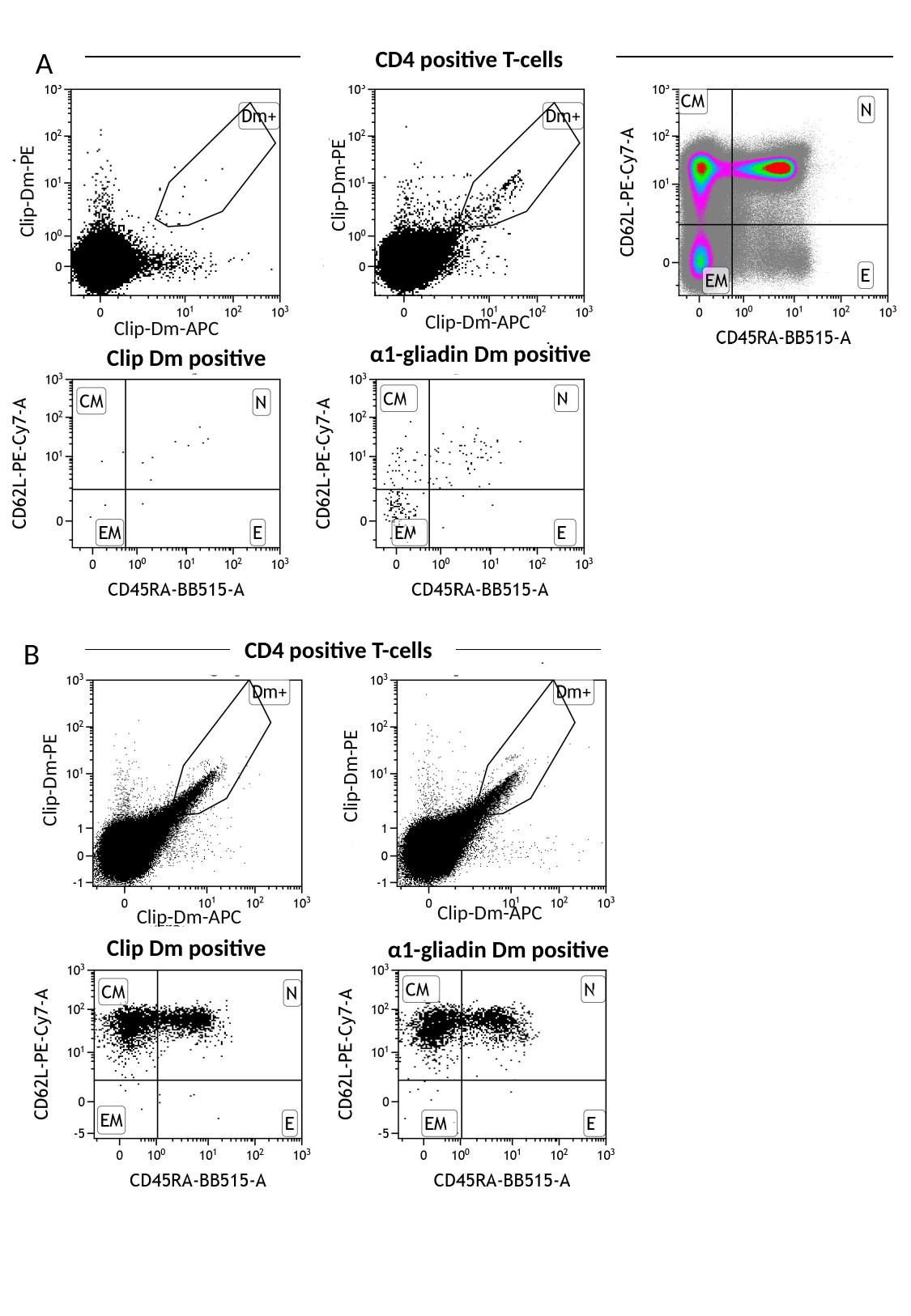

A
CD4 positive T-cells
Clip-Dm-PE
Clip-Dm-PE
Clip-Dm-APC
Clip-Dm-APC
α1-gliadin Dm positive
Clip Dm positive
B
CD4 positive T-cells
Clip-Dm-PE
Clip-Dm-PE
Clip-Dm-APC
Clip-Dm-APC
Clip Dm positive
α1-gliadin Dm positive
