# Supplementary figures and images for: A laboratory test to detect gliadin-specific CD4+ T-cells for difficult to diagnose celiac disease
Source: J Transl Autoimmun. 2025 Jul 24;11:100301. doi: 10.1016/j.jtauto.2025.100301 (PMC12329281; doi:10.1016/j.jtauto.2025.100301)

## Slide 1
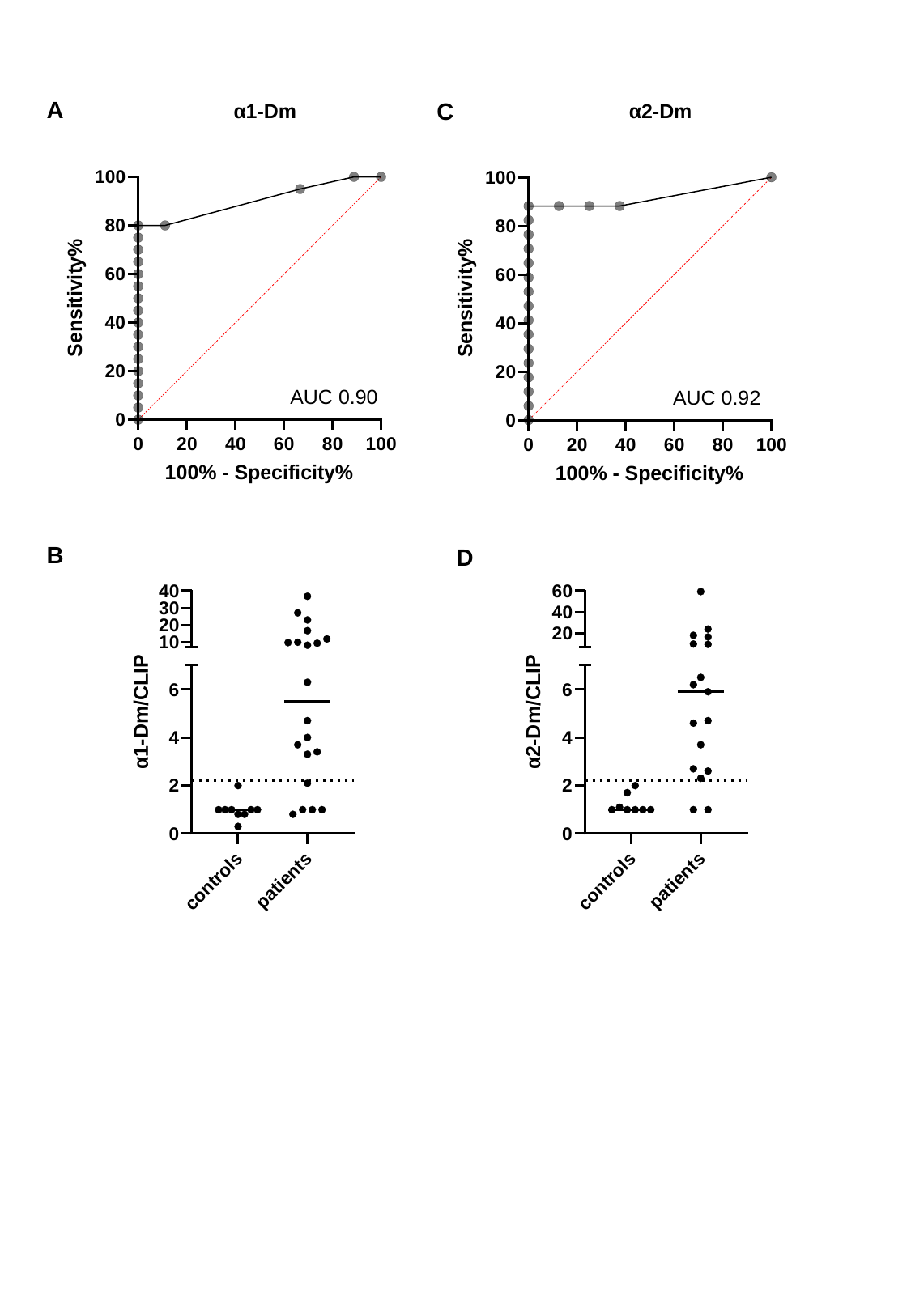

A
C
α1-Dm
α2-Dm
AUC 0.90
AUC 0.92
B
D

Supplement: Multimedia component 6 — Fig. S6Receiver operating characteristic (ROC) curve analysis of (a) and (b) α1- and (c) and (d) α2-Dm:CLIP-Dm ratios in CD4+ TEM for diagnosis of CD [file mmc6.pptx]

## Slide 1
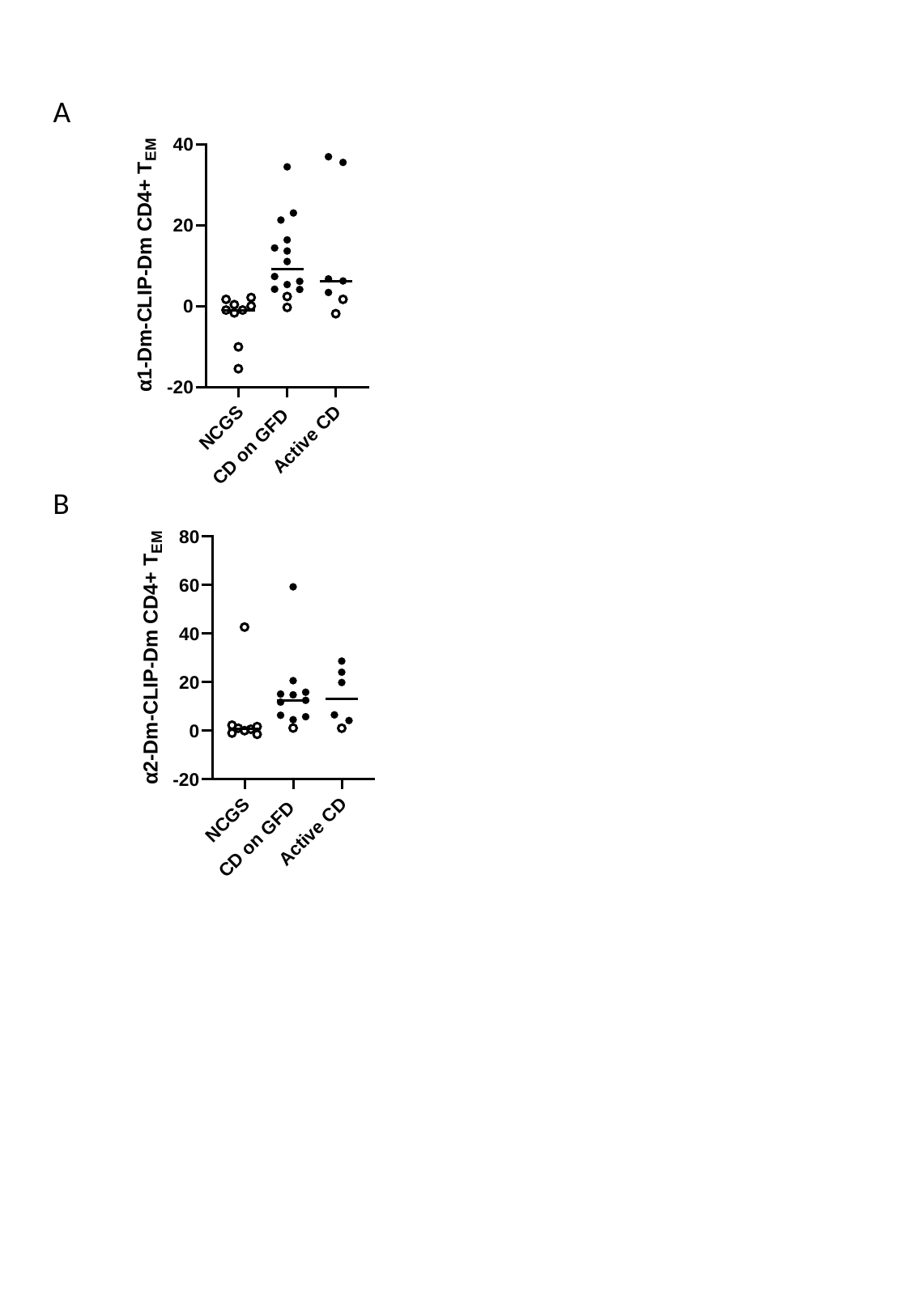

A
B

Supplement: Multimedia component 7 — Fig. S7The number of (a) α1-Dm positive and (b) α2-Dm positive CD4+ TEM, corrected for background by subtracting the number of CLIP-Dm positive CD4+ TEM. Open symbols illustrate individual patients with a ratio below the threshold [file mmc7.pptx]
